# VPAC2 receptor agonist BAY 55-9837 increases SMN protein levels and moderates disease phenotype in severe spinal muscular atrophy mouse models

**DOI:** 10.1186/1750-1172-9-4

**Published:** 2014-01-09

**Authors:** Jeremiah Hadwen, Duncan MacKenzie, Fahad Shamim, Kevin Mongeon, Martin Holcik, Alex MacKenzie, Faraz Farooq

**Affiliations:** 1 University of Ottawa, Ottawa K1H 8M5, Canada; 2 Children’s Hospital of Eastern Ontario Research Institute, Apoptosis Research Centre, 401 Smyth Road, Ottawa Ontario K1H 8L1, Canada

**Keywords:** p38 pathway, SMN, SMA, VPAC2 receptor agonist, Therapeutics

## Abstract

**Background:**

Spinal Muscular Atrophy (SMA) is one of the most common inherited causes of infant death and is caused by the loss of functional survival motor neuron (SMN) protein due to mutations or deletion in the *SMN1* gene. One of the treatment strategies for SMA is to induce the expression of the protein from the homologous *SMN2* gene, a rescuing paralog for SMA.

**Methods and results:**

Here we demonstrate the promise of pharmacological modulation of *SMN2* gene by BAY 55-9837, an agonist of the vasoactive intestinal peptide receptor 2 (VPAC2), a member of G protein coupled receptor family. Treatment with BAY 55-9837 lead to induction of SMN protein levels via activation of MAPK14 or p38 pathway *in vitro*. Importantly, BAY 55-9837 also ameliorated disease phenotype in severe SMA mouse models.

**Conclusion:**

Our findings suggest the VPAC2 pathway is a potential SMA therapeutic target.

## Background

Spinal muscular atrophy (SMA), is an untreatable recessive neuromuscular disorder; with an incidence of 1:11000, it is a leading genetic cause of pediatric death [1]. The loss of lower motor neurons from the ventral horn of spinal cord is the major pathological feature of the disease and results in generalized weakness, progressive muscle loss and respiratory failure [2]. SMA is caused by the pathologic reduction in survival of motor neuron (SMN) protein levels due to deletions and mutations in *SMN1* gene [3]. Although the complete loss of SMN protein is embryonically lethal, the presence of the paralogous *SMN2*, a result of a recent duplication event, which produces a limited full length SMN mRNA (~10%) precludes this outcome in humans [4,5]. Thus all SMA patients have 2 or more copies of *SMN2* gene which in part compensates for the loss of *SMN1* gene. The inverse correlation between the severity of the disease phenotype and copy number of *SMN2*, both confirms the gene’s disease modifying function and has made the induction of *SMN2* a common SMA therapeutic goal. In this regard we have showed the post transcriptional stabilization of SMN mRNA through activation of p38 pathway leads increased SMN levels [6]. We have recently reported that the activation of the p38 pathway through celecoxib upregulates SMN protein levels and can ameliorate disease phenotype in SMA mouse model [7]. In this regard, Vasoactive intestinal peptide receptor 2 (VPAC2), a member of G protein coupled receptor family when activated has been reported to activate p38 pathway *in vivo*[8,9].

We thus decided to assess the blood brain barrier (BBB) penetrant VPAC2 receptor agonist BAY 55-9837 for its potential SMA therapeutic utility. We show here that BAY 55-9837 conferred an increase in SMN protein levels via p38 activation in human neuronal cells. Importantly, we show that treatment with BAY 55-9837 also increases brain and spinal cord SMN protein levels as well as improving disease phenotype and survival in a severe SMA mouse model. Our results provide further evidence that p38 MAPK pathway activators act as potential therapeutic compounds for the treatment of SMA and identify the VPAC pathway as one means of achieving such activation.

## Methods

### Animals

All protocols were approved by Animal Care and Veterinary Services (ACVS) and Ethics board of University of Ottawa. All experiments were carried out in accordance with the Canadian Institute of Health Research (CIHR) Guidebook and ACVS legislation. CD-1 mice were obtained from Charles River Laboratory. The original breeding pair of heterozygous SMA∆7 (*mSmn*+/-, *hSMN2*+/+, *hSMNΔ7*+/+; stock# 005025), Taiwanese mice (*Smn1*^*tm1Hung*^ Tg(SMN2)2Hung/J; stock# 005058) and heterozygous *Smn* knock-out mice (*Smn*^+/-^) on the FVB background were provided by the Jackson Laboratory. The animals were maintained in an air-conditioned ventilated animal facility. Survival, righting time and weight were monitored daily as described by Aviva *et al*[10].

### BAY 55-9837 administration

BAY 55-9837 was diluted in PBS/dH_2_O and administered through IP injection using a 30-gauge needle (0.2 mg/kg dose). Control animals received equal volumes of vehicle alone. SMA∆7 and Taiw/Jax SMA mice were genotyped at P0 and BAY 55-9837 treatment was started from P1. Animals were sacrificed within twenty four hours of the final dose.

### Reagents

BAY 55-9837 was purchased from Tocris Bioscience. p38 inhibitor SB239063 was purchased from Sigma. The antibodies used in this study were SMN/Smn (BD Transduction Laboratories), Actin (Abcam), Tubulin (Abcam), Phospho-p38 (Cell signalling) and Total p38 (Cell signalling).

### Cell culture and drug treatment conditions

Human neuron-committed teratocarcinoma (NT2), mouse motor neuron derived (MN-1) cells and SMA type I patient fibroblasts were maintained in standard conditions (37°C in a 5% CO_2_ humidified atmosphere) in Dulbecco’s modified Eagle medium (DMEM) supplemented with 10% fetal calf serum (FCS), 1% antibiotics (100 units/ml penicillin-streptomycin) and 2 mM glutamate.

NT2 or MN-1 cells were seeded in 6 well plates (5 × 10^5^cells/well) and treated 24 h later with BAY 55-9837 (0.25 μM) for 24h. For time course experiment, NT2 cells were seeded in 6 well plates (5 × 10^5^cells/well) and treated 24h later with BAY 55-9837 (0.25 μM) for up to 24h. For p38 inhibitor treatment, NT2 were seeded in 6 well plates (5 × 10^5^cells/well) and pre-treated with p38 inhibitor SB239063 for 2 h followed by BAY 55-9837 treatment (0.25 μM) for 24 h.

### Western blot analysis

Cells were washed 2 times with 1 ml PBS (1X) and lysed in 150μl RIPA buffer containing 10 mg/ml each of aprotinin, PMSF and leupeptin (all from Sigma), 5 mM β-Glycerolphosphate, 50 mM NaF and 0.2 μM sodium orthovanadate for 30 min at 4°C, followed by centrifugation at 13 000 × g for 30 min; supernatants were then collected and kept frozen at -20°C. Tissue samples were homogenized in 0.5 ml RIPA (10 mg/ml each of aprotinin, PMSF and leupeptin) and then sonicated for 15 seconds. Total protein concentrations were determined by Bradford protein assay using a Bio-Rad protein assay kit. For western blot analysis, protein samples were separated by 11% SDS-PAGE. Proteins were subsequently transferred onto nitrocellulose membrane and incubated in blocking solution (PBS, 5% non-fat milk, 0.2% Tween-20) for 1 h at room temperature followed by overnight incubation with primary antibody at 4°C at the dilution prescribed by the manufacturer. Membranes were washed with PBS-T (PBS, and 0.2% Tween-20) 3 times followed by incubation with secondary antibody (anti-mouse or rabbit, Cell signalling) for 1 h at room temperature. Antibody complexes were visualized by autoradiography using the ECL Plus and ECL western blotting detection systems (GE Healthcare). Quantification was performed by scanning the autoradiographs and signal intensities were determined by densitometric analysis using the ImageJ program.

### Primer sequences

#### For genotyping

Genotyping was performed as previously described by Aviva *et al*[10] for SMAΔ7 mice using the following primers

##### *mSmn* WT

Forward: 5′-TCTGTGTTCGTGCGTGGTGACTTT-3′.

Reverse 1877: 5′-CCCACCACCTAAGAAAGCCTCAAT-3′.

##### Lac Z

Forward: 5′-CCAACTTAATCGCCTTGCAGCACA-3′.

Reverse: 5′-AAGCGAGTGGCAACATGGAAATCG 3′.

##### Human *SMN2 trans*gene

Forward: 5′-CAAACACCTGGTATGGTCAGTC-3′.

Reverse: 5′-GCACCACTGCACAACAGCCTG-3′.

Product sizes:

*mSMN*: 372 bp

Lac Z: 626 bp

*SMN2* transgene: 250 bp

Genotyping for Taiwanese SMA mice was performed as previously described by Riessland *et al*[11] using recommended primers.

### Statistical methods

GraphPad Prism software package was used for the Kaplan–Meier survival analysis. The log-rank test was used and survival curves were considered significantly different at P < 0.05.

Data in figures (histograms, points on graphs) are mean values with the standard error mean (SEM) shown as error bars. The Student’s two-tail *t* test was used to test for statistical differences between samples and were considered significantly different at *P* < 0.05.

## Results and discussion

SMA is a frequently severe neurodegenerative disease which most frequently affects children; many of them do not survive beyond the first few years of life. Although there is no effective therapy for SMA, one translational approach is to induce the paralogous gene *SMN2*. This results in the production of more SMN protein, which can partially compensate for the loss of *SMN1* gene and to moderate the disease phenotype.

### BAY 55-9837 treatment upregulates SMN protein *in vitro*

**Figure 1:**
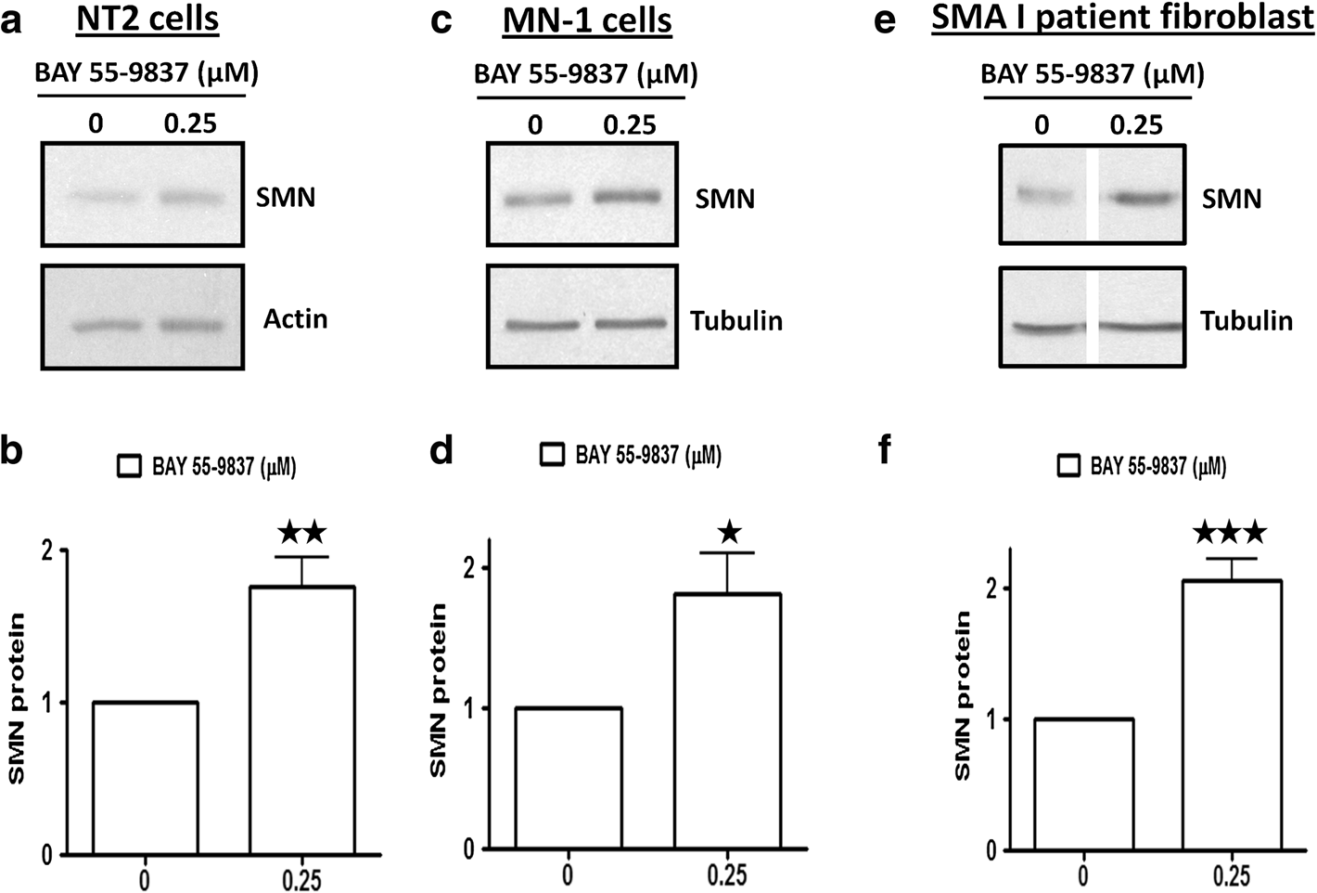
**BAY 55-9837 treatment upregulates SMN protein *in vitro*.** NT2, MN-1 and SMA I patient fibroblasts were treated with BAY 55-9837 (0.25 μM) and then harvested at 24 hours for western blot analyses. **(a)** Representative western blots showing the effect of BAY 55-9837 on SMN protein in NT2 cells. **(b)** Densitometric quantification of SMN protein relative to Actin (the ratio at control treatment was set as 1; mean + SEM (bars) of six independent experiments) are shown for NT2 cells. **(c)** Representative western blot showing effect of BAY 55-9837 on SMN protein in MN-1 cells. **(d)** Densitometric quantification of SMN protein relative to Tubulin (the ratio at control treatment was set as 1; mean + SEM (bars) of three independent experiments) are shown for MN-1 cells. **(e)** Representative western blots showing the effect of BAY 55-9837 on SMN protein in SMA I patient fibroblasts (all lanes were run on the same gel but were non-contiguous). **(f)** Densitometric quantification of SMN protein relative to Tubulin (the ratio at control treatment was set as 1; mean + SEM (bars) of five independent experiments) are shown for SMA I patient fibroblasts. *P < 0.05; **P < 0.01; ***P < 0.001, t-test.

VPAC2 receptor activation has been reported to activate the p38 kinase pathway [8,9] which, in turn, we have shown to stabilize SMN transcript and increase SMN protein level [6]. In order to assess the potential of VPAC-2 receptor activation in the regulation of *SMN* gene expression; human NT2, mouse MN-1 cells and SMA I patient fibroblasts were treated with VPAC2 receptor agonist BAY 55-9837 (25 μM) for 24 h and subsequently harvested for western blot analysis. SMN protein levels were found to be increased by ~2 fold in all cell lines upon treatment with BAY 55-9837 (Figure 1a-f). These results were encouraging in that the increase in SMN protein levels was observed in both neuronal cell lines and patient fibroblasts suggesting that the induction was not specific to a given cell line.

### BAY 55-9837 conferred increase in SMN protein levels is mediated by p38 MAPK activation

**Figure 2:**
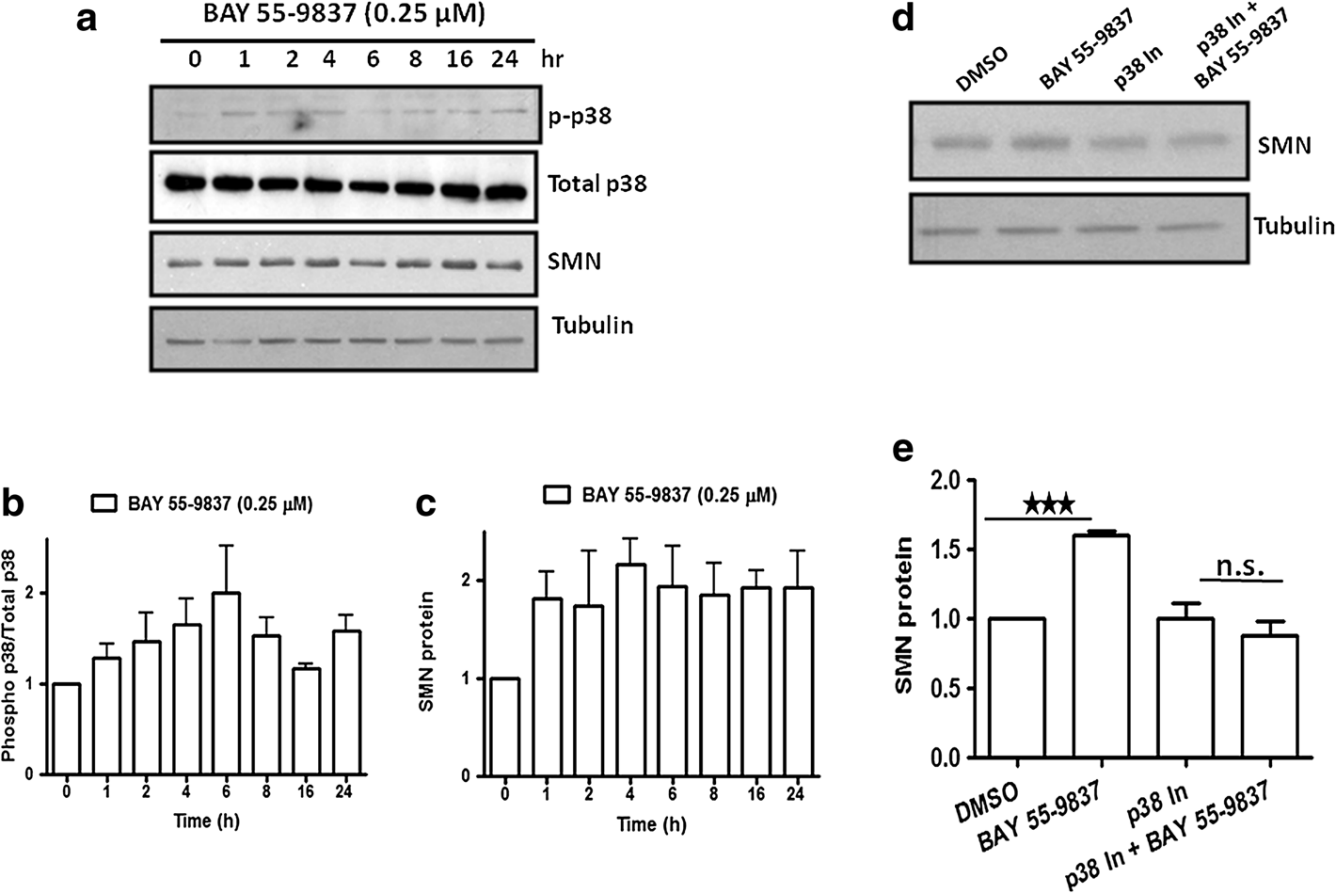
**BAY 55-9837 treatment increases SMN expression via p38 MAPK pathway. (a)** Representative western blot showing activation of p38 MAPK pathway upon BAY 55-9837 treatment in NT2 cells. NT2 cells were treated with BAY 55-9837 at indicated times and then harvested for western blot analysis. Activation of p38 pathway by BAY 55-9837 leads to an increase in SMN protein. **(b)** Densitometric quantification of phospho-p38 relative to total-p38 (the ratio at control treatment was set as 1; mean + SEM (bars) of three independent experiments) are shown for NT2 cells. **(c)** Densitometric quantification of SMN protein relative to Tubulin (the ratio at control treatment was set as 1; mean + SEM (bars) of three independent experiments) are shown for NT2 cells. **(d)** Representative western blots showing the effect of p38 inhibition on BAY 55-9837-induced increase in SMN protein. p38 inhibitor (SB-239580) blocked the BAY 55-9837-induced increase in SMN protein in NT2 cells. NT2 cells were treated with SB-239580 (p38 In; 3 μM) for 2 h followed by treatment with BAY 55-9837 for 24 h and than harvested for western blot analysis. **(e)** Densitometric quantification of SMN protein relative to Tubulin (the ratio at control treatment was set as 1; mean + SEM (bars) of three independent experiments) are shown for NT2 cells showing the effect of p38 inhibition on BAY 55-9837-induced increase in SMN protein. *P < 0.05; **P < 0.01, t-test.

The p38 MAPK pathway regulates a number of cellular process including post-transcriptional stabilization of a distinct class of mRNAs that contain AU rich elements (ARE) mapping to their 3′ UTRs [12-16]. This class of mRNA includes that encoded by *SMN2*[14]; we have previously reported that p38 MAPK increases *SMN* protein expression by virtue of the binding of HUR protein to *SMN2* 3′UTR [6]. The VPAC2 receptor agonist Ro 25-1553 has been previously shown to activate the p38 MAPK pathway [9]; we wished to confirm that BAY 55-9837 could elicit the same p38 activation and that this was underlying the observed SMN protein induction. NT2 cells were therefore treated with BAY 55-9837 and then harvested; western blot analysis at the indicated time intervals revealed within one hour an increase in the ratio of phosphorylated/ total p38 protein (up to 24 hrs after BAY 55-9837 treatment) consistent with p38 activation (Figure 2a-b). p38 MAPK activation was concurrent with the increase in SMN protein levels in NT2 cells (Figure 2a & c). To confirm the role of p38 in the observed SMN protein induction, NT2 cells were pre-treated with the p38 inhibiting agent SB-239063 [17] for 2 h prior to treatment with BAY 55-9837 for 24 h. Western blot analysis revealed that p38 inhibition effectively blocked the BAY 55-9837-mediated increase in SMN protein (Figure 2d & e). These results demonstrate that activation of p38 pathway presumable through binding of VPAC2 receptor agonist to its receptor confers the increase in SMN protein levels observed upon BAY 55-9837 treatment. This result is consistent with our previous observation of increased SMN protein levels conferred by the p38 MAPK activating small compounds anisomycin and celecoxib [6,7].

### BAY 55-9837 treatment upregulates SMN protein levels *in vivo*

In order confirm that BAY 55-9837-mediated SMN protein induction extends to the *in vivo* setting, a dose finding study was initiated. CD-1 mice were given daily intraperitoneal (IP) BAY 55-9837 injections for 5 days 0.02, 0.2 and 2 mg/kg and brain and spinal cord samples then isolated for western blot analysis. Increased SMN protein levels were observed both in brain (Additional file 1: Figure S1a & b) and spinal cord samples (Additional file 1: Figure S1c & d) following BAY 55-9837 treatment with the greatest induction (~2 fold), seen at 0.2 mg/kg dose in CD-1 mice.

**Figure 3:**
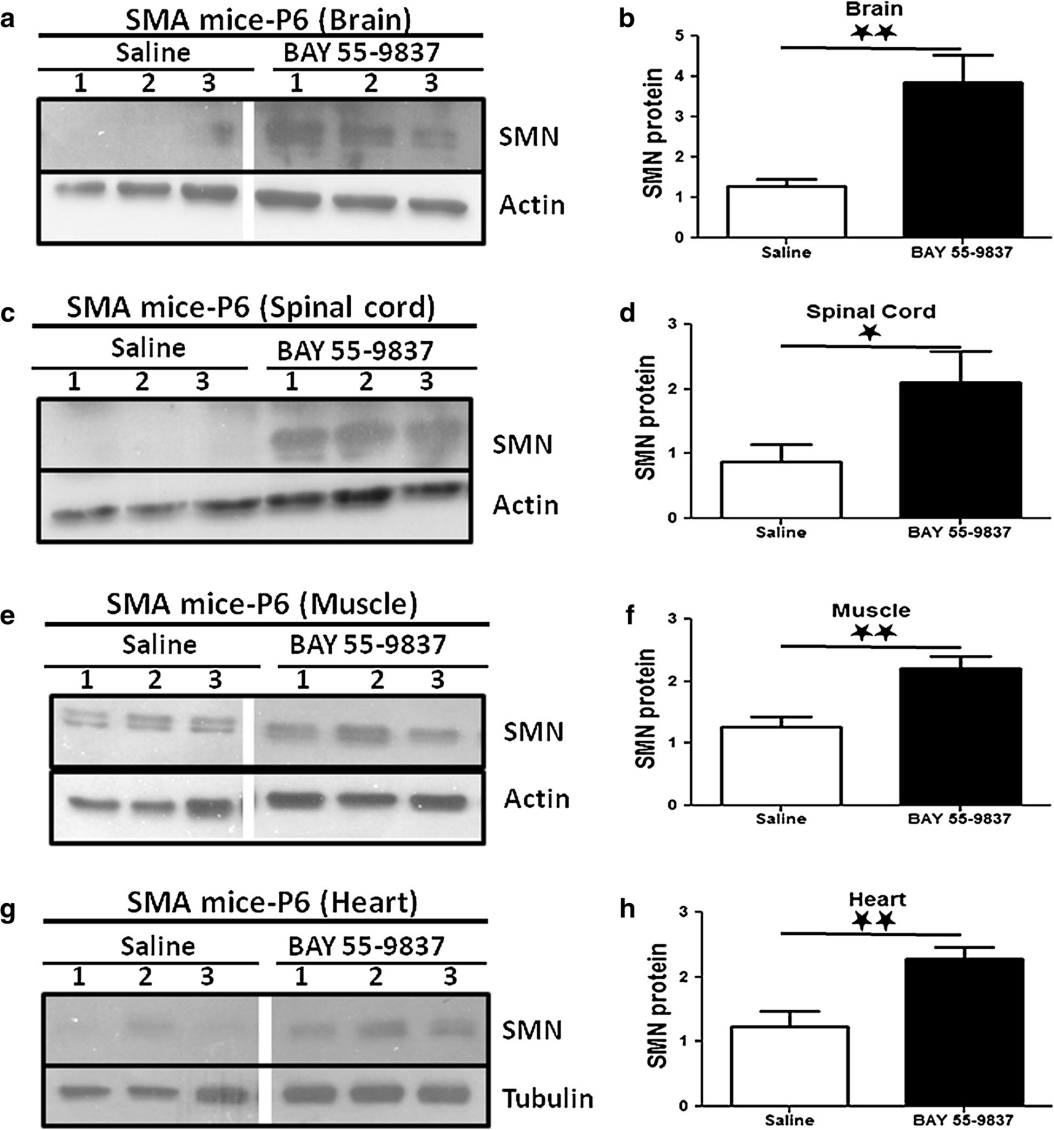
**BAY 55-9837 upregulates SMN protein in SMA mouse model.** SMA∆7 mice were treated daily with saline or BAY 55-9837 (200 μg/kg) from P1 for 6 days, then sacrificed at P7. Brain, spinal cord,skeletal muscle and heart tissues were harvested for western blot analysis. Representative western blots showing effect of BAY 55-9837 on SMN protein in brain **(a)**, spinal cord **(c)**, muscle **(e)** and heart **(g)** samples of SMA∆7 mice treated with Saline (control, lane 1, 2 & 3) or BAY 55-9837 (treatment lane 1, 2 & 3 respectively) (each lane represents individual animal; all lanes were run on the same gel but were non-contiguous). Densitometric quantification of SMN relative to Actin/Tubulin [mean + SEM (bars)] is shown for brain (**b**; n=11), spinal cord (**d**; n=6), muscle (**f**; n=11) and heart (**h**; n=11) samples. *P < 0.05; **P < 0.01; t-test.

We next explored the impact of BAY 55-9837-induced SMN upregulation in a severe mouse model of the disease (SMA∆7 mouse; *mSmn*-/-;*hSMN2*+/+, *hSMNΔ7*+/+ [18]). SMA∆7 mice were given 0.2 mg/kg BAY 55-9837 IP injections twice daily from P1 until P6. Mice were euthanized 24 hours after their last treatment and brain, spinal cord, muscle and heart samples then harvested for western blot analysis. Mice treated with BAY 55-9837 demonstrated an approximate doubling in *SMN2-*derived full length SMN protein levels in all tissues except brain where an approximate quadrupling of SMN protein was observed when compared with vehicle treated animals (Figure 3). In keeping with these results, VPAC2 receptors are expressed in CNS as well as in peripheral tissues [19-25]. The most modest (although still significant) induction of SMN protein was seen in muscle tissues compared to saline treated SMA mice, a possible result of the comparatively low amount of p38 transcript in SMA I muscle compared with normal muscle [26].

### BAY 55-9837 treatment improves disease phenotype in SMA mice model

**Figure 4:**
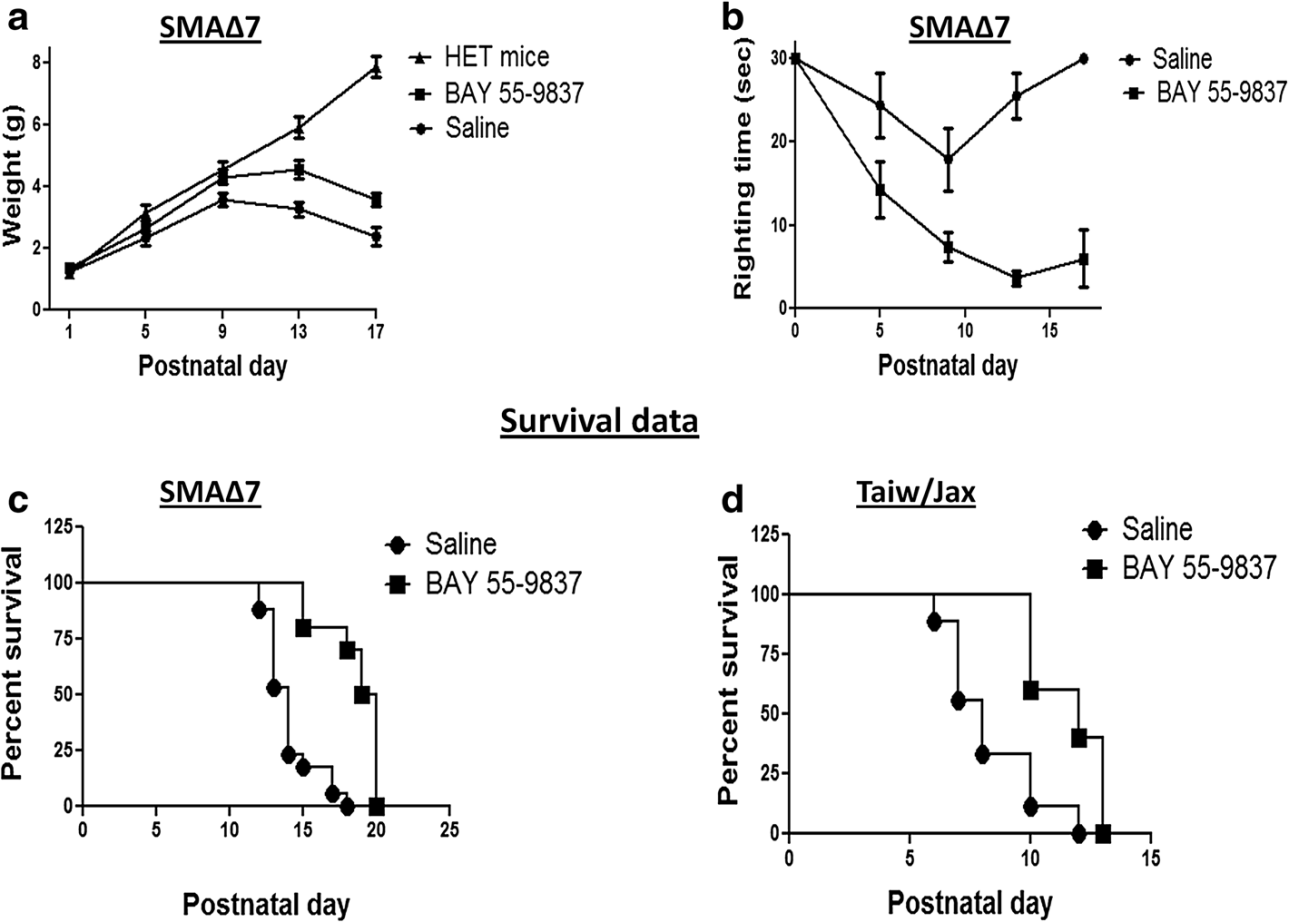
**BAY 55-9837 ameliorates disease phenotype and increases survival of SMA mouse models.** SMA∆7 mice were treated daily with intraperitoneal injections of BAY 55-9837 (200.0 μg/kg) from P1 onward. **(a)** Weights of SMA∆7 mice treated with BAY 55-9837 (black filled square, n =10) or saline (black filled circle, n =10); weights for heterozygous mice treated with saline (black filled triangle, n =5) are also shown for comparison [mean ± SEM (bars)]. **(b)** Righting times of SMA∆7 mice treated with BAY 55-9837 (black filled square, n = 10) or saline (black filled circle, n =10) [mean ± SEM (bars)]. **(c)** Kaplan-Meier survival curves of SMA∆7 mice treated with BAY 55-9837 (black filled square, n = 10; median survival 19.5 days) or saline (black filled circle, n =17; median survival 14.0 days ). **(d)** Kaplan-Meier survival curves of Taiwanese-SMA mice treated with BAY 55-9837 (black filled square, n = 5; median survival 12.0 days) or saline (black filled circle, n =10; median survival 8.0 days); *P < 0.05; ***P < 0.0001, log-rank test.

We next examined the effect of BAY 55-9837 treatment on SMA∆7 mouse disease phenotype. The SMA∆7 mice are significantly underweight and have reduced motor activity compared to heterozygous and WT littermates. SMA∆7 mice were given twice daily BAY 55-9837 or vehicle IP injections starting at P1; their weight and motor function were assessed daily. SMA∆7 mice treated with BAY 55-9837 showed significant improvement in weight gain and motor function (as assessed by righting time), as compared to vehicle-treated SMA∆7 mice (Figure 4a & b).

We also examined the impact of BAY 55-9837 on survival in two different severe SMA mouse models (SMA∆7 [18] and Taiw/Jax-SMA{Cross between Taiwanese mice (*Smn1*^*tm1Hung*^ Tg(SMN2)2Hung/J; stock# 005058) and heterozygous *Smn* knock-out mice (*Smn*^+/-^) [11]}. Significant extension of survival was observed in both mouse models upon treatment with BAY 55-9837 (median survival of 19.5 d for SMA∆7 mice from 14d and 12 days from 8d for Taiw/Jax SMA mice) as compared with vehicle-treated (Figure 4c & d). To account for the treatment effect variability between various laboratories and mouse models, the ratio of median survival of treated to non treated animals was used to assess drug response on survival. With BAY 55-9837 we have achieved a ratio of 1.39 (19.5d/14d for SMA∆7) & 1.5 (12d/8d for Taiw/Jax-SMA) for the two different models. These numbers compare favourably with the other small compounds previously used for SMA treatment such as TSA (1.2; 19d/16d albeit P5 TSA initiation in SMA∆7) [10], SAHA (1.3; 12.9d/9.9d in Taiw/Jax-SMA) [11], celecoxib (1.38; 18d/13d) [7] and Prolactin (1.5; 21d/14d) [27].

SMA is primarily considered as a motor neuron disease and consequently treatment strategies focus on drugs which can cross the blood brain barrier (BBB) to target tissues within central nervous system (CNS). However several recent studies challenge this notion and suggest that SMN has function above and beyond motor neurons and reclassify SMA as a multi-system disorder (including cardiovascular, peripheral necrosis, pancreatic and liver defects) [28-36]. In this regard the widespread presence of the VPAC2 receptor augurs well for this pathway as a therapeutic SMA target [25].

## Conclusion

**Figure 5:**
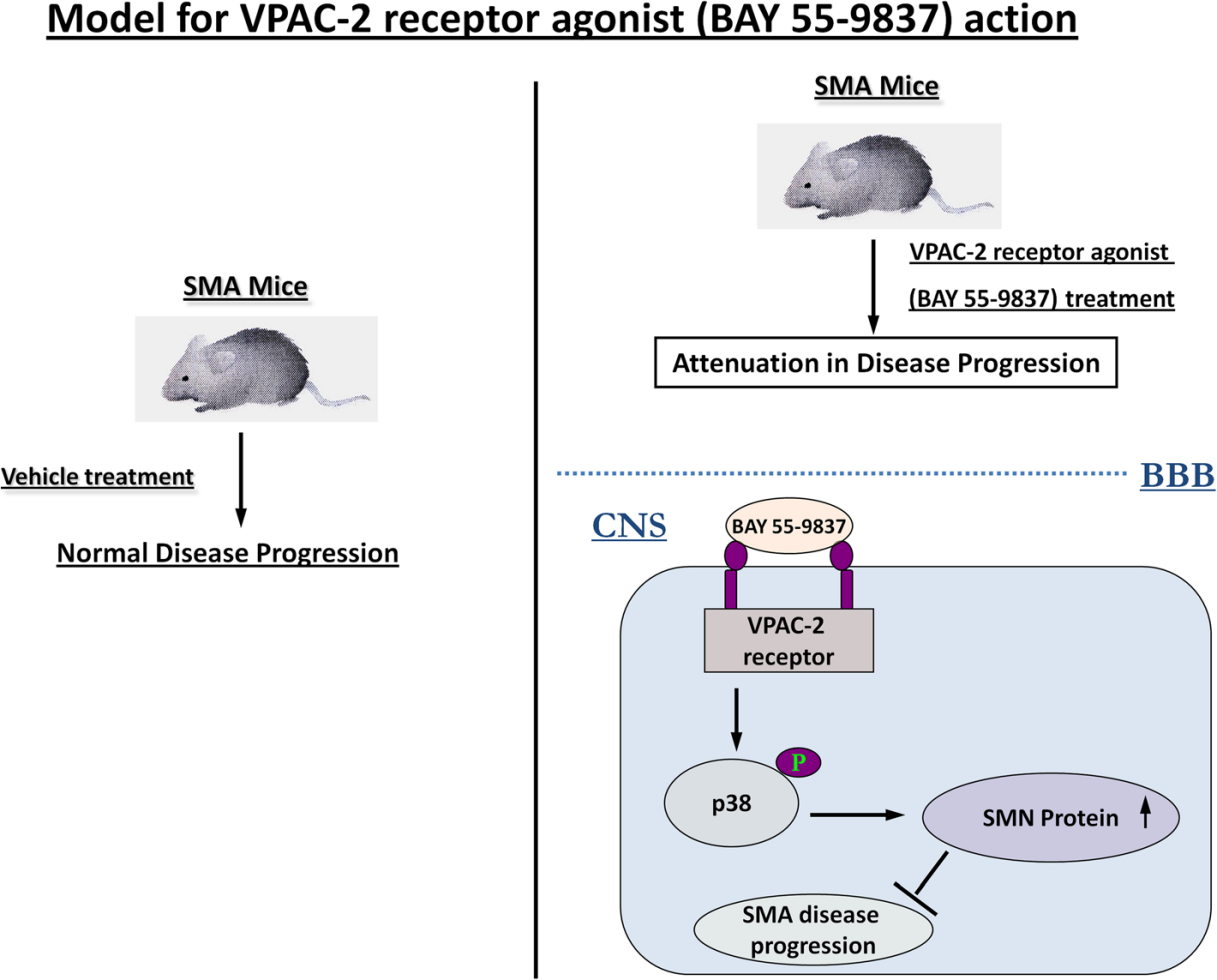
Model for VPAC2 receptor agonist (BAY 55-9837) action.

Re-purposing drugs for distinct disease indications is becoming a more common practice given the approximately 7000 orphan genetic disorders that are estimated to exist. This approach is both cost-effective as well as shortening the path to treatment for significant (and currently untreatable) disorders such as SMA. In the current study, BAY 55-9837 initially developed for the treatment of diabetes [37,38] has been used as a p38 activating compound for the treatment of murine SMA. Our results demonstrate that VPAC2 receptor agonist BAY 55-9837 increases SMN protein levels and attenuates disease progression in two distinct severe SMA mouse models (Figure 5) providing a proof of concept and support for other VPAC2 agonists/p38 activating compounds to be tested as effective SMA therapies. Although the literature on the safety profile of BAY 55-9837 is divided (e.g. 38 and 39), in our experiments we did not observe any adverse effects. Nevertheless further work to obtain comprehensive safety profile for BAY 55-9837 will be beneficial [39]. This study provides a good supportive evidence as well as functional insight how p38 pathway can be targeted for its potential application towards development of therapeutics for SMA.

## Competing interests

The authors declare that they have no competing interests.

## Authors’ contributions

FF designed and executed the experiments, analyzed the data, and wrote the manuscript; JH, and DM assisted with the experiments and analysis of the data; FS and KM assisted with the experiments; MH and AM assisted with analysis and writing. All authors read and approved the final manuscript.

## Supplementary Material

Additional file 1: Figure S1 BAY 55-9837 upregulates Smn protein in wild type mice. 4 weeks old CD-1 wild type mice were treated daily with BAY 55-9837 (2.0, 20.0, 200.0 μg/kg) for 5 days, then sacrificed. Brain and spinal cord tissues were harvested for western blot analysis. (a) Representative western blot showing the effect of BAY 55-9837 on Smn protein in brain samples of CD-1 mice treated with saline (control, lane-1) or BAY 55-9837 (lane 2, 3 & 4 respectively) (n = 3). (b) Densitometric quantification of SMN relative to Tubulin [mean + SEM (bars)] is shown for brain samples. (c) Representative western blot showing the effect of BAY 55-9837 on SMN protein in spinal cord samples of CD-1 mice treated with Saline (control, lane-1) or BAY 55-9837 (lane 2, 3 & 4) (n = 3). (d) Densitometric quantification of SMN relative to Tubulin [mean + SEM (bars)] is shown for spinal cord samples. *P < 0.05, t- test. Click here for file

## References

[B1] Sugarman EA Nagan N Zhu H Akmaev VR Zhou Z Rohlfs EM Flynn K Hendrickson BC Scholl T Sirko-Osadsa DA Allitto BA Pan-ethnic carrier screening and prenatal diagnosis for spinal muscular atrophy: clinical laboratory analysis of >72,400 specimens Eur J Hum Gen 2012 20 27 32 10.1038/ejhg.2011.134 PMC3234503 21811307

[B2] D’Amico A Mercuri E Tiziano FD Bertini E Spinal muscular atrophy Orphanet J Rare Dis 2011 6 71 10.1186/1750-1172-6-71 22047105 PMC3231874

[B3] Lefebvre S Burglen L Reboullet S Clermont O Burlet P Viollet L Benichou B Cruaud C Millasseau P Zeviani M Identification and characterization of a spinal muscular atrophy-determining gene Cell 1995 80 155 165 10.1016/0092-8674(95)90460-3 7813012

[B4] Lorson CL Hahnen E Androphy EJ Wirth B A single nucleotide in the SMN gene regulates splicing and is responsible for spinal muscular atrophy Proc Natl Acad Sci USA 1999 96 6307 6311 10.1073/pnas.96.11.6307 10339583 PMC26877

[B5] Monani UR Lorson CL Parsons DW Prior TW Androphy EJ Burghes AH McPherson JD A single nucleotide difference that alters splicing patterns distinguishes the SMA gene SMN1 from the copy gene SMN2 Hum Mol Genet 1999 8 1177 1183 10.1093/hmg/8.7.1177 10369862

[B6] Farooq F Balabanian S Liu X Holcik M MacKenzie A p38 Mitogen-activated protein kinase stabilizes SMN mRNA through RNA binding protein HuR Hum Mol Genet 2009 18 4035 4045 10.1093/hmg/ddp352 19648294

[B7] Farooq F Abadia-Molina F MacKenzie D Hadwen J Shamim F O’Reilly S Holcik M MacKenzie A Celecoxib increases SMN and survival in a severe spinal muscular atrophy mouse model via p38 pathway activation Hum Mol Genet 2013 22 3415 3424 10.1093/hmg/ddt191 23656793

[B8] Garry EM Delaney A Blackburn-Munro G Dickinson T Moss A Nakalembe I Robertson DC Rosie R Robberecht P Mitchell R Fleetwood-Walker SM Activation of p38 and p42/44 MAP kinase in neuropathic pain: involvement of VPAC2 and NK2 receptors and mediation by spinal glia Mol Cell Neurosci 2005 30 523 537 10.1016/j.mcn.2005.08.016 16202621

[B9] Monaghan TK Mackenzie CJ Plevin R Lutz EM PACAP-38 induces neuronal differentiation of human SH-SY5Y neuroblastoma cells via cAMP-mediated activation of ERK and p38 MAP kinases J Neurochem 2008 104 74 88 17995938 10.1111/j.1471-4159.2007.05018.x PMC2230095

[B10] Avila AM Burnett BG Taye AA Gabanella F Knight MA Hartenstein P Cizman Z Di Prospero NA Pellizzoni L Fischbeck KH Sumner CJ Trichostatin A increases SMN expression and survival in a mouse model of spinal muscular atrophy J Clin Invest 2007 117 659 671 10.1172/JCI29562 17318264 PMC1797603

[B11] Riessland M Ackermann B Forster A Jakubik M Hauke J Garbes L Fritzsche I Mende Y Blumcke I Hahnen E Wirth B SAHA ameliorates the SMA phenotype in two mouse models for spinal muscular atrophy Hum Mol Genet 2010 19 1492 1506 10.1093/hmg/ddq023 20097677

[B12] Brook M Sully G Clark AR Saklatvala J Regulation of tumour necrosis factor alpha mRNA stability by the mitogen-activated protein kinase p38 signalling cascade FEBS Lett 2000 483 57 61 10.1016/S0014-5793(00)02084-6 11033356

[B13] Dean JL Brook M Clark AR Saklatvala J p38 mitogen-activated protein kinase regulates cyclooxygenase-2 mRNA stability and transcription in lipopolysaccharide-treated human monocytes J Biol Chem 1999 274 264 269 10.1074/jbc.274.1.264 9867839

[B14] Frevel MA Bakheet T Silva AM Hissong JG Khabar KS Williams BR p38 Mitogen-activated protein kinase-dependent and -independent signaling of mRNA stability of AU-rich element-containing transcripts Mol Cell Biol 2003 23 425 436 10.1128/MCB.23.2.425-436.2003 12509443 PMC151534

[B15] Rutault K Hazzalin CA Mahadevan LC Combinations of ERK and p38 MAPK inhibitors ablate tumor necrosis factor-alpha (TNF-alpha ) mRNA induction. Evidence for selective destabilization of TNF-alpha transcripts J Biol Chem 2001 276 6666 6674 10.1074/jbc.M005486200 11076936

[B16] Wang SW Pawlowski J Wathen ST Kinney SD Lichenstein HS Manthey CL Cytokine mRNA decay is accelerated by an inhibitor of p38-mitogen-activated protein kinase Inflamm Res 1999 48 533 538 10.1007/s000110050499 10563470

[B17] Legos JJ Erhardt JA White RF Lenhard SC Chandra S Parsons AA Tuma RF Barone FC SB 239063, a novel p38 inhibitor, attenuates early neuronal injury following ischemia Brain Res 2001 892 70 77 10.1016/S0006-8993(00)03228-5 11172750

[B18] Le TT Pham LT Butchbach ME Zhang HL Monani UR Coovert DD Gavrilina TO Xing L Bassell GJ Burghes AH SMNDelta7, the major product of the centromeric survival motor neuron (SMN2) gene, extends survival in mice with spinal muscular atrophy and associates with full-length SMN Hum Mol Genet 2005 14 845 857 10.1093/hmg/ddi078 15703193

[B19] Basille M Cartier D Vaudry D Lihrmann I Fournier A Freger P Gallo-Payet N Vaudry H Gonzalez B Localization and characterization of pituitary adenylate cyclase-activating polypeptide receptors in the human cerebellum during development J Comp Neurol 2006 496 468 478 10.1002/cne.20934 16572459

[B20] Harmar AJ Sheward WJ Morrison CF Waser B Gugger M Reubi JC Distribution of the VPAC2 receptor in peripheral tissues of the mouse Endocrinology 2004 145 1203 1210 10.1210/en.2003-1058 14617572

[B21] Kalamatianos T Kallo I Piggins HD Coen CW Expression of VIP and/or PACAP receptor mRNA in peptide synthesizing cells within the suprachiasmatic nucleus of the rat and in its efferent target sites J Comp Neurol 2004 475 19 35 10.1002/cne.20168 15176082

[B22] Reubi JC In vitro evaluation of VIP/PACAP receptors in healthy and diseased human tissues. Clinical implications Ann N Y Acad Sci 2000 921 1 25 11193811 10.1111/j.1749-6632.2000.tb06946.x

[B23] Reubi JC Laderach U Waser B Gebbers JO Robberecht P Laissue JA Vasoactive intestinal peptide/pituitary adenylate cyclase-activating peptide receptor subtypes in human tumors and their tissues of origin Cancer Res 2000 60 3105 3112 10850463

[B24] Schulz S Rocken C Mawrin C Weise W Hollt V Schulz S Immunocytochemical identification of VPAC1, VPAC2, and PAC1 receptors in normal and neoplastic human tissues with subtype-specific antibodies Clin Cancer Res 2004 10 8235 8242 10.1158/1078-0432.CCR-04-0939 15623599

[B25] Sherwood NM Krueckl SL McRory JE The origin and function of the pituitary adenylate cyclase-activating polypeptide (PACAP)/glucagon superfamily Endocr Rev 2000 21 619 670 11133067 10.1210/edrv.21.6.0414

[B26] Millino C Fanin M Vettori A Laveder P Mostacciuolo ML Angelini C Lanfranchi G Different atrophy-hypertrophy transcription pathways in muscles affected by severe and mild spinal muscular atrophy BMC Med 2009 7 14 10.1186/1741-7015-7-14 19351384 PMC2676312

[B27] Farooq F Molina FA Hadwen J MacKenzie D Witherspoon L Osmond M Holcik M MacKenzie A Prolactin increases SMN expression and survival in a mouse model of severe spinal muscular atrophy via the STAT5 pathway J Clin Invest 2011 121 3042 3050 10.1172/JCI46276 21785216 PMC3148738

[B28] Araujo A Araujo M Swoboda KJ Vascular perfusion abnormalities in infants with spinal muscular atrophy J Pediatr 2009 155 292 294 10.1016/j.jpeds.2009.01.071 19619755 PMC3250227

[B29] Bevan AK Hutchinson KR Foust KD Braun L McGovern VL Schmelzer L Ward JG Petruska JC Lucchesi PA Burghes AH Kaspar BK Early heart failure in the SMNDelta7 model of spinal muscular atrophy and correction by postnatal scAAV9-SMN delivery Hum Mol Genet 2010 19 3895 3905 10.1093/hmg/ddq300 20639395 PMC2947399

[B30] Bowerman M Swoboda KJ Michalski JP Wang GS Reeks C Beauvais A Murphy K Woulfe J Screaton RA Scott FW Kothary R Glucose metabolism and pancreatic defects in spinal muscular atrophy Ann Neurol 2012 72 256 268 10.1002/ana.23582 22926856 PMC4334584

[B31] Gogliotti RG Quinlan KA Barlow CB Heier CR Heckman CJ Didonato CJ Motor neuron rescue in spinal muscular atrophy mice demonstrates that sensory-motor defects are a consequence, not a cause, of motor neuron dysfunction J Neurosci 2012 32 3818 3829 10.1523/JNEUROSCI.5775-11.2012 22423102 PMC3679185

[B32] Heier CR Satta R Lutz C DiDonato CJ Arrhythmia and cardiac defects are a feature of spinal muscular atrophy model mice Hum Mol Genet 2010 19 3906 3918 10.1093/hmg/ddq330 20693262 PMC2947406

[B33] Hua Y Sahashi K Rigo F Hung G Horev G Bennett CF Krainer AR Peripheral SMN restoration is essential for long-term rescue of a severe spinal muscular atrophy mouse model Nature 2011 478 123 126 10.1038/nature10485 21979052 PMC3191865

[B34] Martinez TL Kong L Wang X Osborne MA Crowder ME Van Meerbeke JP Xu X Davis C Wooley J Goldhamer DJ Survival motor neuron protein in motor neurons determines synaptic integrity in spinal muscular atrophy J Neurosci 2012 32 8703 8715 10.1523/JNEUROSCI.0204-12.2012 22723710 PMC3462658

[B35] Rudnik-Schoneborn S Vogelgesang S Armbrust S Graul-Neumann L Fusch C Zerres K Digital necroses and vascular thrombosis in severe spinal muscular atrophy Muscle Nerve 2010 42 144 147 10.1002/mus.21654 20583119

[B36] Shababi M Habibi J Yang HT Vale SM Sewell WA Lorson CL Cardiac defects contribute to the pathology of spinal muscular atrophy models Hum Mol Genet 2010 19 4059 4071 10.1093/hmg/ddq329 20696672

[B37] Pan CQ Li F Tom I Wang W Dumas M Froland W Yung SL Li Y Roczniak S Claus TH Engineering novel VPAC2-selective agonists with improved stability and glucose-lowering activity in vivo J Pharmacol Exp Ther 2007 320 900 906 17110523 10.1124/jpet.106.112276

[B38] Tsutsumi M Claus TH Liang Y Li Y Yang L Zhu J Dela Cruz F Peng X Chen H Yung SL A potent and highly selective VPAC2 agonist enhances glucose-induced insulin release and glucose disposal: a potential therapy for type 2 diabetes Diabetes 2002 51 1453 1460 10.2337/diabetes.51.5.1453 11978642

[B39] Darsalia V Mansouri S Wolbert P Barde S Sjoholm A Patrone C The specific VPAC2 agonist Bay 55-9837 increases neuronal damage and hemorrhagic transformation after stroke in type 2 diabetic rats Neuropeptides 2012 47 133 137 22981158 10.1016/j.npep.2012.08.008

