# Supplementary figures and images for: VPAC2 receptor agonist BAY 55-9837 increases SMN protein levels and moderates disease phenotype in severe spinal muscular atrophy mouse models
Source: Orphanet J Rare Dis. 2014 Jan 9;9:4. doi: 10.1186/1750-1172-9-4 (PMC3895859; doi:10.1186/1750-1172-9-4)

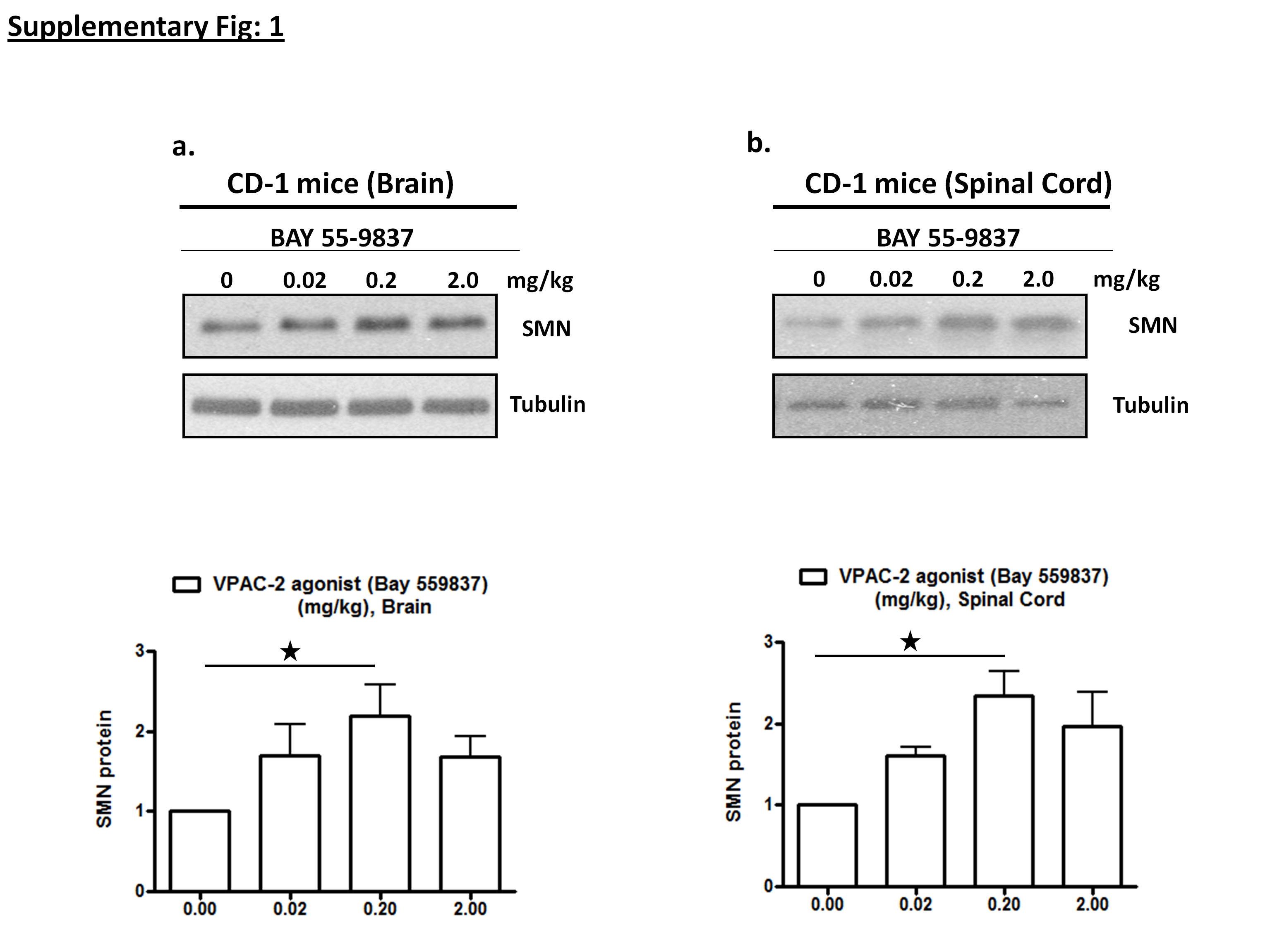

Supplement: Additional file 1: Figure S1 — BAY 55-9837 upregulates Smn protein in wild type mice. 4 weeks old CD-1 wild type mice were treated daily with BAY 55-9837 (2.0, 20.0, 200.0 μg/kg) for 5 days, then sacrificed. Brain and spinal cord tissues were harvested for western blot analysis. (a) Representative western blot showing the effect of BAY 55-9837 on Smn protein in brain samples of CD-1 mice treated with saline (control, lane-1) or BAY 55-9837 (lane 2, 3 & 4 respectively) (n = 3). (b) Densitometric quantification of SMN relative to Tubulin [mean + SEM (bars)] is shown for brain samples. (c) Representative western blot showing the effect of BAY 55-9837 on SMN protein in spinal cord samples of CD-1 mice treated with Saline (control, lane-1) or BAY 55-9837 (lane 2, 3 & 4) (n = 3). (d) Densitometric quantification of SMN relative to Tubulin [mean + SEM (bars)] is shown for spinal cord samples. *P < 0.05, t- test. [file 1750-1172-9-4-S1.tiff]
